# Does Moderate Coronary Stenosis Affect the Fate of the Left Internal Thoracic Artery Graft?

**DOI:** 10.21470/1678-9741-2018-0001

**Published:** 2018

**Authors:** Aytac Caliskan, Ertekin Utku Unal, Emre Kubat, Bahadir Aytekin, Basak Soran Turkcan, Erman Sureyya Kiris, Muharrem Tola, Hakki Zafer Iscan

**Affiliations:** 1 Cigli District Training Hospital, Cardiovascular Surgery, Izmir, Turkey.; 2 Turkey Yuksek Ihtisas Training and Research Hospital, Cardiovascular Surgery Ankara, Turkey.; 3 Karabuk Training and Research Hospital, Cardiovascular Surgery, Karabuk, Turkey.; 4 Turkey Yuksek Ihtisas Training and Research Hospital, Radiology, Ankara,Turkey.

**Keywords:** Coronary Angiography, Coronary Artery Bypass, Ultrasonography, Internal Mammary-Coronary Artery Anastomosis, Coronary Stenosis

## Abstract

**Introduction:**

In this study we try to observe the fate of the left internal thoracic artery grafts that were bypassed to left anterior descending artery with moderate stenosis identified with fractional flow reserve (FFR) technique. Doppler ultrasonography was chosen as a noninvasive screening method.

**Methods:**

A total of 30 patients who underwent coronary artery bypass grafting depending on results of the fractional flow reserve between January 2007 and January 2012, were subjected to transthoracic color Doppler ultrasonographic evaluation irrespective of the presence of symptoms, and the presence of a systolic-diastolic flow pattern was investigated using the supraclavicular approach.

**Results:**

The left internal thoracic artery graft was found to be functional in 63.3% of patients within a mean period of 35.1±19.7 months between coronary bypass and color Doppler ultrasonography. This period was found to be 29.4±19.6 months in the functional graft group, and 44.7±16.6 months in the dysfunctional graft group (*P*=0.046). Preoperative complaints of angina were reported to fall from 88.9% to 16.7% in the functional graft group, when compared to the postoperative period (*P*<0.001), but fell from 90.9% to 36.4% in the dysfunctional graft group (*P*=0.034).

**Conclusion:**

Functional left internal thoracic artery graft rates of the study population were found to be lower than the studies reported in the literature.

**Table:** 

Abbreviations, acronyms & symbols	
**CABG**	**= Coronary artery bypass grafting**
**CAG**	**= Coronary angiography**
**CDUS**	**= Color Doppler ultrasonography**
**FFR**	**= Fractional flow reserve**
**ITAG**	**= Internal thoracic artery grafts**
**LAD**	**= Left anterior descending artery**
**NYHA**	**= New York Heart Association**
**PCI**	**= Percutaneous coronary interventions**

## INTRODUCTION

Results of the interventions to severe coronary artery stenoses are mostly predictable. However, controversy still exists concerning moderate stenoses. Rapid increase in plaque sizes of borderline lesions have been reported, and long periods of nonintervention with these lesions can lead to undesirable cardiac events^[1,2]^. Coronary artery bypass grafting (CABG) to moderately stenotic coronary vessels that were diagnosed by qualitative coronary angiography (CAG), is associated with lower long-term mortality^[3]^. But the success of surgical intervention in moderate stenoses is suggested to be associated with the functional importance of the lesion. Fractional flow reserve (FFR) is the suggested technique to decide the functional importance of a moderate coronary stenosis^[4]^. FFR has a very critical role in a surgeon's decision because a graft that was bypassed to a coronary artery with a functionally insignificant stenosis may be dysfunctional as a result of the competitive flow^[5]^. In this study we try to observe the fate of the left internal thoracic artery grafts (ITAG) that were bypassed to left anterior descending artery (LAD) with moderate stenoses that were identified with FFR technique. The patients were included in the study regardless of their symptom status so we chose color Doppler ultrasonography (CDUS) as a noninvasive screening method. The studies, which have demonstrated that CDUS could detect ITAG patency at a rate of over 90%, had encouraged us to choose CDUS as a screening method^[6,7]^.

## METHODS

### Study Patients

A cross-sectional examination, from the data of 494 patients who were subjected to FFR for LAD between January 2007 and January 2012, was conducted. In our institution, experienced interventional cardiologists and surgeons decide the severity of the lesion mostly by visual assessment, but FFR is performed in case of suspected moderate stenosis. The decision to perform CABG has been made for patients with a FFR value ≤0.80. Patients needing associated valve surgery and emergent CABG (defined as within 24 hours of the index procedure) were excluded. The 128 patients who underwent CABG at our hospital and who could be contacted by phone or by mail were invited for outpatient follow-ups; 30 of these invited patients responded to the calls and came for follow-up controls. Data concerning the variables were obtained from patients' medical records in the automation system, and from patient files. Patients were also examined according to the New York Heart Association (NYHA) functional capacity and the presence of angina, on arrival at the hospital for follow-up. Evaluation of angina was made according to standard guidelines recommendations as 'definite angina', 'probable angina', 'probably not angina' and 'definitely not angina'; and patients in the 'definite angina' and 'probable angina' group were considered as having angina^[8]^. All the patients were subjected to transthoracic echocardiography. Transthoracic CDUS was also performed in all the patients for the assessment of ITAG irrespective of the presence of symptoms. CAG was recommended to all patients who reported postoperative angina complaints during their outpatient follow-up visits.

### Surgical Technique

While preparing the ITAG after median sternotomy in all patients, the great saphenous vein was used as a graft in all patients who were scheduled for bypass in more than one vessel. The ITAG was prepared from the origin of the subclavian artery to the superior epigastric and musculophrenic branches in the sixth intercostal space, using low-current electrocautery and hemoclips in the side branches, releasing it together with the pedicle and wrapping it with papaverine gas tampon. ITAG was harvested in all cases by senior residents.

Standard cannulation was made from the ascending aorta and the right atrium (two-stage venous cannula). The targeted heparinization was accomplished at 300-400 IU/kg and a target activated clotting time is 480 seconds. Surgery was performed under mild hypothermia (34-35ºC). We did not observe any off-pump operation in study population.

The ITAG of all patients in the study population was anastomosed to the LAD. No medication was administered to the patients after surgery to prevent bleeding. All patients were transferred to the postoperative intensive care unit and placed on a mechanical ventilator for a few hours for follow-up.

### CDUS Technique

All CDUS conducted for the visualization of the ITAG was performed using the Logiq 7 (Medical Systems, Milwaukee, WI, USA) Doppler ultrasonographic device and the 3-7 MHz multi-frequency linear probe. Examination of the ITAG was made using the CDUS by the supraclavicular approach, to visualize the subclavian artery towards the origin of the vertebral artery in the vertical plane. The presence of a cystolo-diastolic flow pattern was then investigated by visualizing the origin of the internal thoracic artery in the form of a tubular structure with the probe rotated caudally 90 degrees clockwise. Patients, whose cystolo-diastolic flow pattern in ITAG could not be examined, were considered to have dysfunctional grafts.

### Statistical Analysis

Continuous numerical variables are expressed as "mean ± standard deviation (SD)" of descriptive statistical analysis. On the other hand, the cut-off and categorized data are expressed as "number" and "percentage (%)". The "Chi-square test" and the "Fisher test" were used to compare categorized variables. Independent variables which were inconsistent with normal distribution were analyzed using the nonparametric "Mann-Whitney U test". The difference between inter-group preoperative and postoperative variables was obtained using the "Wilcoxon test". On the other hand, the time-dependent functional ITAG rate was evaluated using "Kaplan-Meier analysis". The "*P* value" was determined as "α=0.05" in all data analyses for the assessment of statistical significance level. Data were analyzed using the "IBM SPSS Statistics Version 15.0" packet program.

## RESULTS

Basic demographic and clinical characteristics of the 30 patients who form the study population are shown in Table 1.

**Table 1:** Basic demographic and intraoperative characteristics of the patients.

Characteristics of the patients		n	%	Mean ± SD
Gender	Female	5	16.7	
	Male	25	83.3	
Age (years)				63.6±9.6
Diabetes mellitus		4	13.3	
Hypertension		21	70.0	
Peripheral artery disease		2	6.7	
Hyperlipidemia		14	46.7	
Smoking		10	33.3	
Carotid stenosis		1	3.3	
CKD		3	10.0	
SVE		__	__	
COPD		1	3.3	
FFR value				0.69±0.06
EF (%)				54.4±7.9
CPB		30	100.0	
The number of distal coronary bypasses				2.10±0.88
Patients who underwent single vessel coronary artery bypass		10	33.3	
Patients who underwent two or more vessel coronary artery bypass		20	66.7	

CKD=chronic kidney disease; COPD=chronic obstructive pulmonary disease; CPB=cardiopulmonary bypass; EF=ejection fraction; FFR=fractional flow reserve; SD=standard deviation; SVE=cerebrovascular event

Preoperative and postoperative distribution and comparison of symptoms and functional capacities of patients are seen in Table 2. No statistically significant difference was found between the preoperative and postoperative functional capacities of the patients (*P*=0.059). However, patients were reported to have improved from their angina during the postoperative follow-ups (*P*<0.001). Angina complaints have been reduced significantly in patients with single-vessel bypass, as well as in those who underwent coronary artery bypass grafting in two or more vessels (*P*=0.008 and *P*=0.001, respectively).

**Table 2:** Preoperative and postoperative distribution and comparison of patient symptoms and functional capacity values.

Characteristics		n	%	*P* value
Preoperative FC	NYHA 1	18	60.0	
	NYHA 2	7	23.3	
	NYHA 3	5	16.7	0.059
Postoperative FC	NYHA 1	21	70.0	
	NYHA 2	9	30.0	
Preoperative angina		26	89.7	
Postoperative angina		7	24.1	<0.001

FC=functional capacity; NYHA=New York Heart Association

Apart from the CABG, three (10%) patients were subjected to additional intervention with one patient undergoing septal myectomy, one patient carotid endarterectomy, while one patient also underwent repair of femoral artery pseudoaneurysm.

The median FFR value was found to be 0.70 (range: 0.56-0.80). Only two patients were found to be in the 0.75-0.80 range.

The mean period of postoperative stay in the intensive care unit was found to be 1.8±3.1 days for all patients, whereas the postoperative period for hospitalization was reported as 6.3±4.1 days. The mean period between CABG and CDUS was found to be 35.1±19.7 months.

Results from CDUS conducted during outpatient follow-up visits demonstrated that the ITAG was functional in 63.3% of the patients (19 patients). Patients were then divided into two groups (functional and dysfunctional) according to assessment of ITAG, and the statistically significant difference between the groups evaluated (Table 3). A significant difference was reported between the two groups with regards only to the duration between CABG and CDUS. This duration was found to be 29.4±19.6 months in the group with functional ITAG, and 44.7±16.6 months in the dysfunctional group (*P*=0.046).

**Table 3:** Comparison of demographic and clinical characteristics of patients with functional and dysfunctional ITAG on color CDUS.

		Functional		Dysfunctional		
Characteristics		n (%)	Mean ± SS	n (%)	Mean± SD	*P* value
Gender	Female	2 (10.5)		3 (27.3)		0.327
	Male	17 (89.5)		8 (72.7)		
Age (years)			63.4±10.4		64±8.6	0.800
Diabetes mellitus		4 (21.1)		__		0.268
Hypertension		13 (68.4)		8 (72.7)		0.804
Peripheral artery disease		1 (5.3)		1 (9.1)		1.000
Hyperlipidemia		8 (42.1)		6 (54.5)		0.510
Smoking		7 (36.8)		3 (27.3)		0.592
Carotid stenosis		1 (5.3)		__		1.000
CKD		2 (10.5)		1 (9.1)		1.000
COPD		1 (5.3)		__		1.000
FFR value			0.68±0.06		0.72±0.04	0.310
EF (%)			53.8±9.3		55.4±4.7	0.767
Additional procedure		3 (15.3)		__		0.279
Coronary bypass number			2.05±0.97		2.18±0.75	0.800
Preoperative angina		16 (88.9)		10 (90.9)		1.000
Postoperative angina		3 (16.7)		4 (36.4)		0.375
Postoperative CKD		1 (5.3)		__		1.000
Postoperative MI		1 (5.3)		__		1.000
Postoperative low cardiac output		1 (5.3)		__		1.000
Postoperative others		2 (10.5)		__		0.520
Postoperative stay in ICU (days)			2.1±3.8		1.4±0.9	0.902
Postoperative hospitalization (days)			6.5±5.1		6.0±1.2	0.359
Duration between CABG and CDUS (months)			29.4±19.6		44.7±16.6	0.046

CABG=coronary artery bypass grafting; CDUS=color Doppler ultrasonography; CKD=chronic kidney disease; COPD=chronic obstructive pulmonary disease, EF=ejection fraction; FC=functional capacity; FFR=fractional flow reserve; ICU=intensive care unit, ITAG: internal thoracic artery graft; MI=myocardial infarction; NYHA=New York Heart Association; SD=standard deviation

There was no significant difference between the two groups with regards to the preoperative and postoperative functional capacities (ITAG functional group *P*=0.053; ITAG dysfunctional group *P=*0.655).

Four of the patients who were found to be consistent with postoperative angina accepted to undergo CAG. As a result, no statistically significant comparison was performed.

Preoperative complaints of angina were reported to fall from 88.9% to 16.7% in the functional ITAG group, when compared to the postoperative period (*P*<0.001), whereas the complaints fell from 90.9% to 36.4% in the dysfunctional ITAG group (*P*=0.034).

**Fig. 1:**
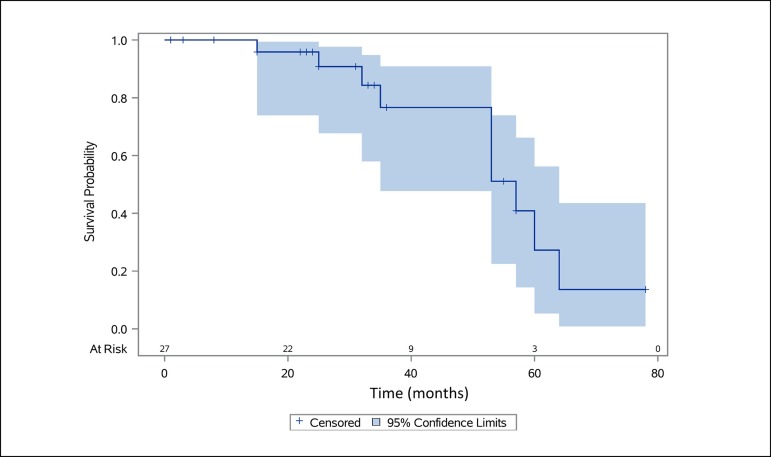
5 year functional left ITAG rates with 95% CI and patients at risk according to Kaplan Meier analysis.

The functional ITAG rates of the patients according to CDUS are shown in Figure 1. Accordingly, the two-year functional graft rates were found to be 90.8%, whereas the three-year functional graft rates were 76.6%.

## DISCUSSION

About 63.3% of the ITAG of the patients who underwent CABG according to FFR values, were found to be functional during a mean postoperative period of 35.1±19.7 months. Functional graft rates were found to be lower than the known patency rates of the ITAG. In the dysfunctional ITAG group the period between CABG and CDUS was found to be significantly longer than that observed in the functional ITAG group.

Moderate coronary stenoses are currently being treated according to their functional significance. Non-intervention for moderate stenoses, considered to be functionally insignificant, has demonstrated to alter survival and the incidence of angina^[9]^. Uneventful survival rate in cases with percutaneous coronary interventions (PCI) performed according to FFR guidance has shown to be higher than in procedures determined by CAG alone, and that FFR reduces the number of vessels involved with, and the cost of PCI^[10]^. These findings are in line with another study which shows that surgical revascularization of moderate lesions according to their functional significance does not increase unwanted cardiac events despite reducing the number of anastomoses^[11]^. These results may be due to the fact that the patency of grafts for coronary stenoses, considered as functionally severe, is higher than that of those considered to be non-severe^[12]^.

ITAG is normally a conduit with a 10-year patency rate of 95%^[13]^. The fact that our study population consisted of patients with moderate stenosis suggests that the low functional ITAG rates (63.3%) may be due to native coronary blood flow. This flow in native vessels is more pronounced in vessels with moderate stenosis than in those with severe stenosis. Moderate target vessel stenoses have shown to be an independent risk factor for arterial graft dysfunction and can particularly cause grafts to become dysfunctional due to competitive flow, reducing the three-year patency rates to less than 90%^[14,15]^. Due to the fact that arterial grafts reduce disease progression and also ensure regression of existing stenosis^[16]^, improvement in native coronary flow may be another reason for dysfunctional ITAG in patients with moderate LAD stenosis as observed in our study population. The flow in the native coronary artery may prevent graft function when it is providing adequate perfusion to the myocardium distal to the stenosis. This sufficient flow in the native vasculature may have rendered patients to remain asymptomatic.

ITAG which could not be assessed with CDUS in our study were considered as being dysfunctional. Literature studies show that the grafts of approximately 10% of patients could not be evaluated with CDUS, and at least 60% of these grafts were detected as being patent on CAG^[6]^. Similarly, one of the reasons why the functional ITAG rate was below the expected level can be attributed to such limitations in the imaging procedure.

The small sample size of our study may be the most important limiting factor. The period between CABG and CDUS are not homogeneous in the study population. Also study results could be affected by the heterogeneity of surgical technique or even surgeons, the diameter of coronary arteries as well as the severity of FFR values. The FFR values in the grey zone, which is considered to be within the range of 0.75-0.80, were reported in only two patients; hence, a comparison could not be performed according to lesion severity. There is another heterogeneity in the number of diseased and bypassed vessels. Results obtained show that there was a significant decrease in postoperative angina complaints in patients with single-vessel bypass, as well as in those who were subjected to bypass grafting in two or more vessels (*P*=0.008 and *P*=0.001). The ITAG was used only at the LAD position in all of the patients and CDUS was used for visualizing only the functional status of the ITAG. Occlusion of saphenous venous grafts of coronary stenosis other than LAD may cause ischemia in some parts of the myocardium, leading to observable symptoms, while saphenous grafts of vessels which are a source of preoperative pain may remain patent, leading to asymptomatic features in the patients. Finally, evaluation of graft function was performed with CDUS; however, results could not be compared to CAG which is considered to be the golden standard.

## CONCLUSION

This study may give us an idea that grafting of moderate coronary stenoses may negatively affect graft patency rates. In order to assess the safety of determining the hemodynamic importance of moderate lesions by the FFR technique, studies with adequate and homogenous follow-up periods, involving a larger study population, and whose CDUS results can be confirmed by CAG, are required so as to obtain more definite results.

**Table:** 

**Authors’ roles & responsibilities**	
AC	Substantial contributions to the conception or design of the work; or the acquisition, analysis, or interpretation of data for the work; final approval of the version to be published
EUU	Substantial contributions to the conception or design of the work; or the acquisition, analysis, or interpretation of data for the work; final approval of the version to be published
EK	Drafting the work or revising it critically for important intellectual content; final approval of the version to be published
BA	Final approval of the version to be published
BST	Substantial contributions to the conception or design of the work; or the acquisition, analysis, or interpretation of data for the work; final approval of the version to be published
ESK	Substantial contributions to the conception or design of the work; or the acquisition, analysis, or interpretation of data for the work; final approval of the version to be published
MT	Performed the echocardiographic evaluation; final approval of the version to be published
HZI	Drafting the work or revising it critically for important intellectual content; final approval of the version to be published
